# Piezotronic effect in AlGaN/AlN/GaN heterojunction nanowires used as a flexible strain sensor

**DOI:** 10.3762/bjnano.11.166

**Published:** 2020-12-10

**Authors:** Jianqi Dong, Liang Chen, Yuqing Yang, Xingfu Wang

**Affiliations:** 1 Institute of Semiconductor Science and Technology, South China Normal University, Guangzhou 510631, China; 2 School of Physics and Optoelectronic Engineering, Guangdong University of Technology, Guangzhou 510006, China

**Keywords:** AlGaN/AlN/GaN nanowires, flexible, piezotronic effect, strain sensors, strain tests, top-down method

## Abstract

1D semiconductor nanowires (NWs) have been extensively studied in recent years due to the predominant mechanical flexibility caused by a large surface-to-volume ratio and unique electrical and optical properties induced by the 1D quantum confinement effect. Herein, we use a top-down two-step preparation method to synthesize AlGaN/AlN/GaN heterojunction NWs with controllable size. A single NW is transferred to a flexible poly(ethylene terephthalate) substrate and fixed by indium tin oxide electrodes to form an ohmic contact for the strain sensor. An external mechanical stress is introduced to study the performance of the fabricated piezotronic strain sensor. The gauge factor is as high as 30 under compressive or tensile stress, which indicates a high sensitivity of the strain sensor. Periodic strain tests show the high stability and repeatability of the sensor. The working mechanism of the strain sensor is investigated and systematically analyzed under compressive and tensile strain. Here, we describe a strain sensor that shows a great application potential in wearable integrated circuits, in health-monitoring devices, and in artificial intelligence.

## Introduction

Due to the non-centrosymmetric structure of the group-III nitride semiconductor materials (e.g., GaN, AlN, and AlGaN), spontaneous polarization (*P*_sp_) and piezoelectric polarization induced by lattice mismatch (*P*_lm_) are inevitably introduced during the epitaxial growth process [1–3]. Furthermore, the analysis of the internal polarization of the AlGaN/AlN/GaN heterojunction showed the existence of a 2D electron gas (2DEG), which effectively suppresses the degradation of the carrier mobility caused by the scattering at charge impurity centers [4]. This is a widely discussed topic in the field of high electron mobility transistor (HEMT) research [5–6]. In order to further improve the physical properties of a 2DEG and optimize the performance of AlGaN/AlN/GaN-based HEMT devices, piezotronic effects are introduced to adjust the polarization distribution inside the heterojunction [7–8]. The piezotronic effect, described first by Zhong Lin Wang in 2007, is a combination of the piezoelectric effect and the properties of non-centrosymmetric semiconductor materials [9].

1D semiconductor nanowires (NWs) are more suitable candidates for the study of the piezotronic effect than nanofilms or bulk materials since the smaller physical size and larger surface-to-volume ratio of 1D NWs yields superior mechanical properties [4,10]. In addition, 1D semiconductor NWs can increase the electron mobility and achieve the confinement of light based on the 1D quantum confinement effect. Hence, the unique electrical and optical properties of 1D semiconductor NWs have attracted research interest from the field of nanogenerators [11–14] and NW-based strain sensors [15–19]. Strain sensors can convert mechanical deformation into electrical signals. They exhibit a potential for application in health-monitoring and motion-monitoring devices, and in artificial intelligence, for example [20–22]. However, high sensitivity (gauge factor ≥20) is key to detect a very small deformation of a given material [23–24]. Therefore, AlGaN/AlN/GaN NWs with high electron mobility, carrier density, and mechanical flexibility have become good candidates for highly sensitive and flexible strain sensors. In this work, we use a top-down two-step process, including inductively coupled plasma (ICP) dry etching and selective electrochemical (EC) wet etching, to prepare AlGaN/AlN/GaN heterojunction NWs with a controllable size. After the lift-off, a single NW is transferred to a flexible poly(ethylene terephthalate) (PET) substrate and is fixed by indium tin oxide (ITO) electrodes to form an ohmic contact for the strain sensor. Under different compressive and tensile strain values, *I*–*V* characteristic curves of the prepared piezotronic strain sensor show the ability of the sensor to detect strain. The gauge factor is calculated under different strain conditions. It is as high as 30 under either a −1.78% compressive strain or a 1.78% tensile strain, which shows its high sensitivity. Furthermore, the current increases with an increase in the tensile strain and decreases with an increase in the compressive strain along the *c*-axis. The current returns to its original value after release of the mechanical stress. At the same time, the multiple cycles of tensile and compressive strain testing also fully demonstrates the repeatability and stability of the strain sensors. The working principle of strain sensors is illustrated by the analysis of the polarized charge distribution under compressive and tensile strain modes. This work describes the fabrication of a highly sensitive and a highly stable strain sensor based on a new AlGaN/AlN/GaN NW structure, which has a great potential to be applied in wearable integrated circuits, health-monitoring devices, artificial intelligence, among other fields.

## Results and Discussion

**Figure 1:**
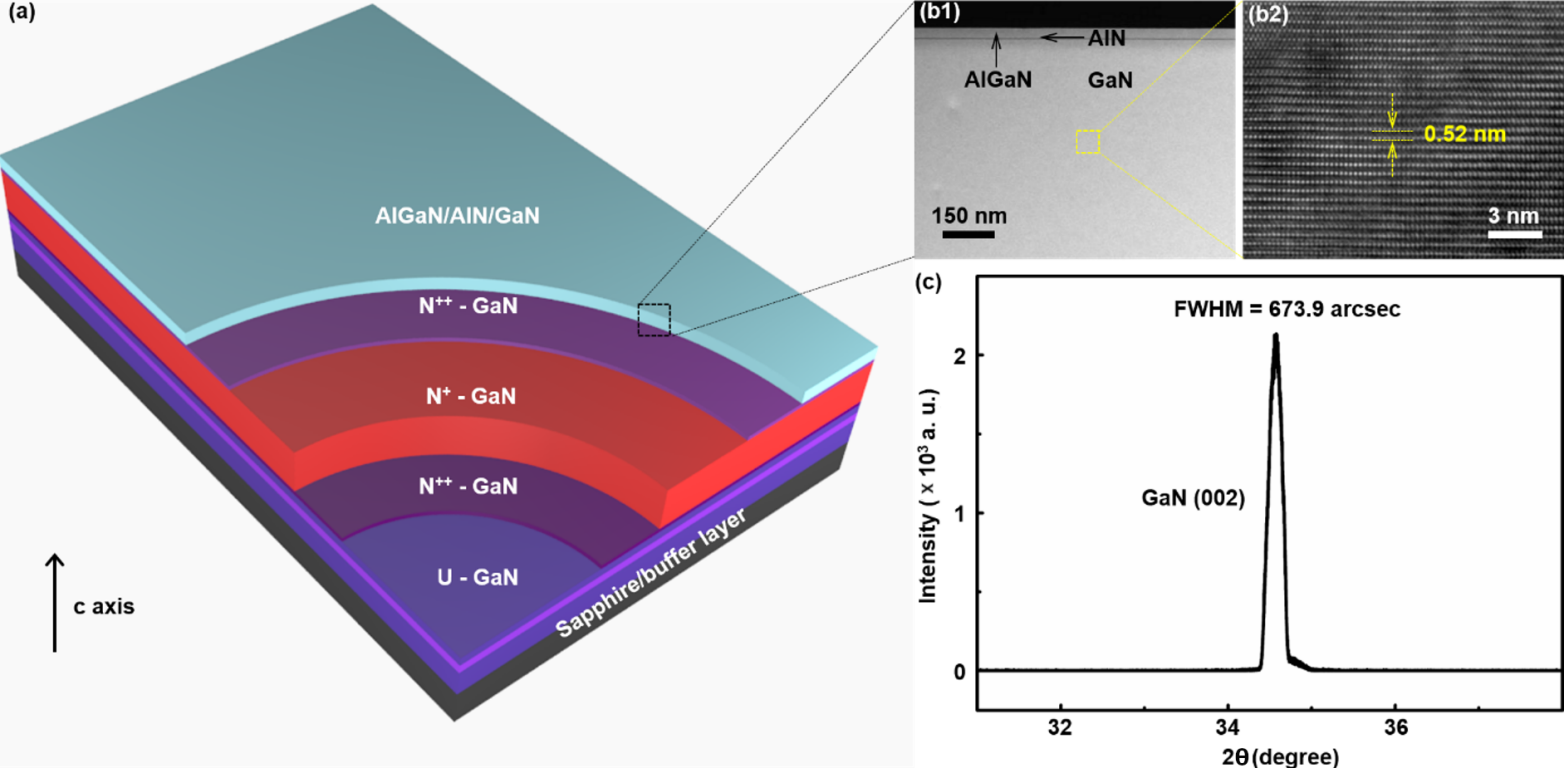
(a) Schematic diagram of the epitaxial structure. (b) STEM image taken of the AlGaN/AlN/GaN heterojunction (left panel, b1) and HRTEM image of the corresponding GaN layer (right panel, b2). (c) XRD 2θ scan of the epitaxial structure in the region of the (002) reflection.

The epitaxial structures used in this study were synthesized by metal-organic chemical vapor deposition (MOCVD), as shown in Figure 1a. First, 1 μm of an unintentionally doped GaN layer was deposited onto a sapphire substrate. Then, 500 nm of n-type GaN, as a current-spreading layer, was deposited to increase the lateral spreading of the current. Next, 500 nm of an unintentionally doped GaN layer was deposited to protect the lower layer during the selective EC etching. A heavily doped GaN (N^+^-GaN) sacrificial layer, sandwiched by two thin N^++^-GaN layers was inserted under the AlGaN/AlN/GaN layer to enhance the conductivity contrast. Detailed structural parameters are shown in the Experimental section.

Figure 1b shows a scanning transmission electron microscopy (STEM) image taken of the AlGaN/AlN/GaN heterojunction (left panel) and a corresponding high-resolution transmission electron microscopy (HRTEM) image of the GaN layer (right panel). It can be seen that there is a clear dividing line (ultrathin AlN layer) between AlGaN and GaN. An interplanar spacing of 0.52 nm was measured in the GaN layer along the [2] direction. Furthermore, an X-ray diffraction (XRD) scan of the epitaxial structure is shown in Figure 1c. The full width at half maximum (FWHM) of the GaN(002) reflection is approx. 673.9 arcsec, which is comparable to that of bulk GaN. These results unambiguously prove that the AlGaN/AlN/GaN heterojunction has an ultrahigh crystal quality, although it was grown onto a heavily doped GaN sacrificial layer.

**Figure 2:**
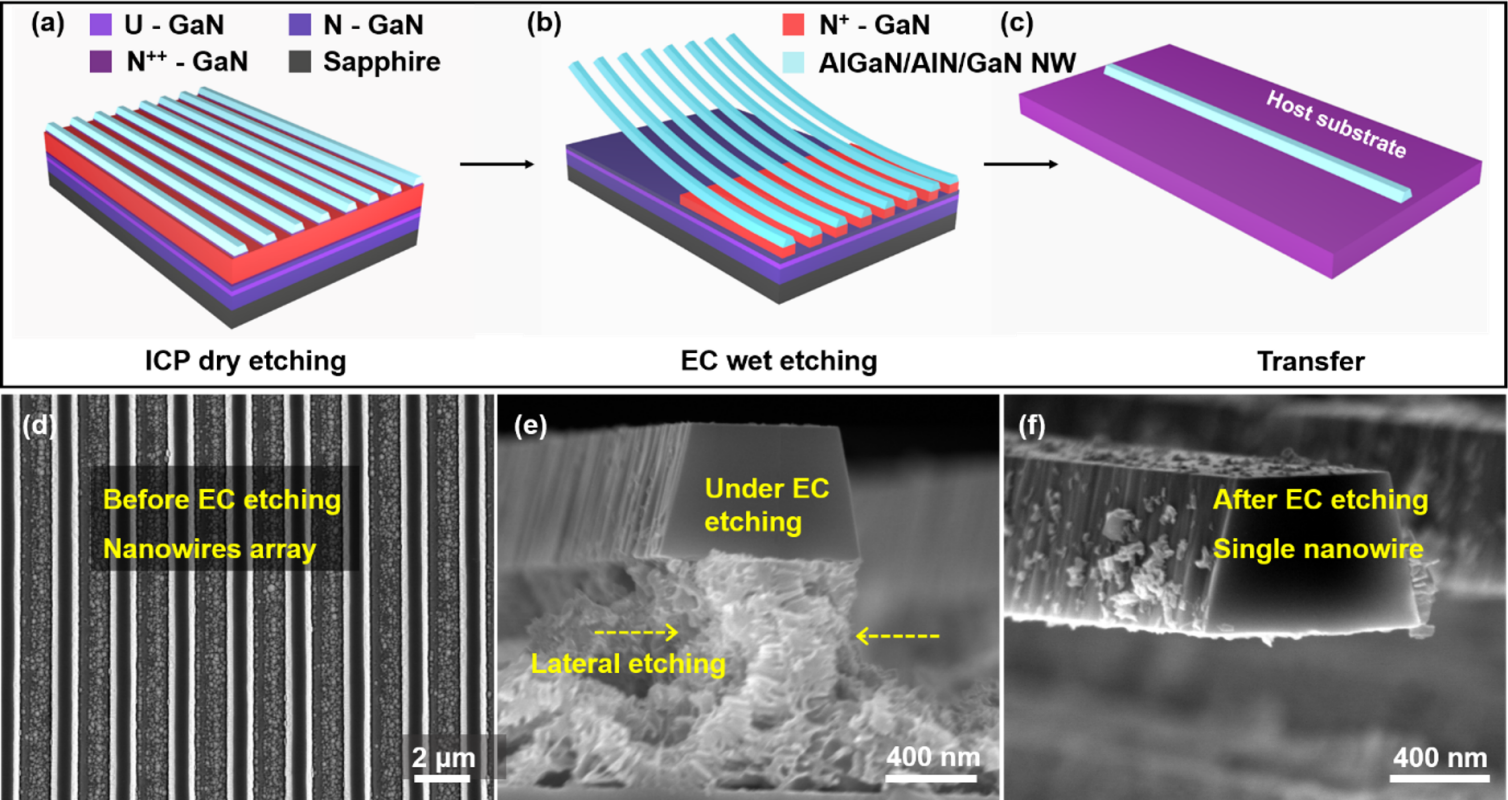
(a) Schematic diagrams after ICP dry etching, (b) during EC wet etching, (c) and of a single nanowire. (d) SEM image of a AlGaN/AlN/GaN heterojunction NW array after ICP dry etching. (e) SEM image during EC etching. (f) SEM image of the released NW after EC etching.

The specific top-down two-step preparation process of freestanding AlGaN/AlN/GaN heterojunction NWs, including isotropic ICP dry etching and selective EC wet etching [25–26], is shown in Figure 2. First, a layer of photoresist was spin coated on the surface of the wafer from MOCVD, and advanced stepper lithography was used to form a striped pattern, which was used as a mask for ICP dry etching. The depth of the ICP dry etching needs to be greater than 1 µm (the thickness of the AlGaN/AlN/GaN heterojunction is 931.5 nm). The purpose is to expose the sacrificial layer for the subsequent EC wet etching. Then, the striped photoresist mask, which covers the surface, was removed with acetone to obtain the structure shown in Figure 2a. The settings for the stripe width (900 nm) and the interval between the stripes (3 μm) were controlled during stepper lithography.

After ICP dry etching, the sample was placed in an electrolytic cell containing 0.3 M oxalic acid solution for EC wet etching. The sacrificial layer (GaN) was dissolved during the EC wet etching to release the upper layer of the AlGaN/AlN/GaN heterojunction NWs (Figure 2b), which was transferred to the host substrate (Figure 2c). Under a suitable applied electric field, the mechanism of EC etching can be described by:


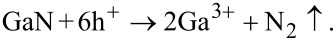


The corresponding SEM images are shown in Figure 2d–f. After ICP dry etching, a regular stripe array was formed (Figure 2d) and the shape of the NWs was controlled in advance during stepper lithography. Because of its high conductivity, the heavily doped GaN preferentially reacts with the oxalic acid solution in an applied electric field. The lateral etching rates on both sides of N^+^-GaN were approximately the same during EC wet etching (Figure 2e). Finally, a single NW was obtained to prepare the strain sensor (Figure 2f).

**Figure 3:**
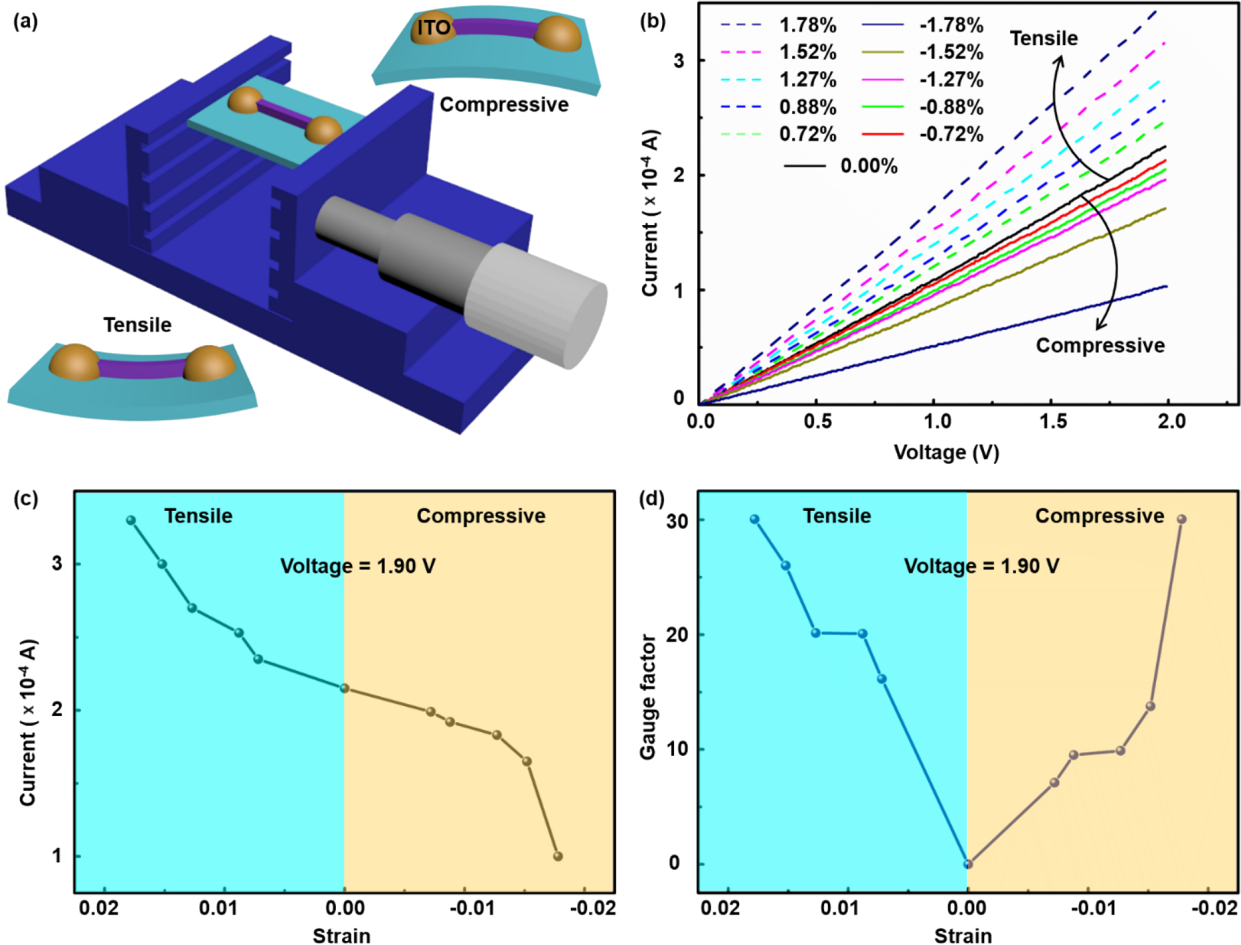
(a) Experimental setup showing the stress application on the AlGaN/AlN/GaN heterojunction NW-based strain sensor. (b) *I*–*V* characteristic curves at different compressive and tensile strain values along the *c*-axis. (c) The relation between current and strain at a bias voltage of 1.9 V. (d) The relation between gauge factor and strain at a bias voltage of 1.9 V.

The ITO transparent electrodes were patterned and deposited onto the two ends of the AlGaN/AlN/GaN heterojunction NWs to eliminate the impact of the Schottky barrier. In addition, they fixed the NW to the flexible PET substrate. A schematic of the equipment (electric one-dimensional translation stage) for different values of compressive or tensile stress applied along the *c*-axis is shown in Figure 3a. The typical *I*–*V* curves were obtained for different strain values (Figure 3b). It can be clearly seen that, as the tensile strain increases along the *c*-axis, the current at a given voltage gradually increases. Conversely, when the compressive strain increases, the current at a given voltage gradually decreases. The mechanism regarding this behavior will be explained below. A characteristic strain–current curve was extracted from the *I*–*V* curves at a bias voltage of 1.9 V, as shown in Figure 3c. The results unambiguously show that there is a positive correlation between strain and current. Taking the current in the free state as the reference, the current increased by 53.55% at 1.78% tensile strain, whereas it decreased by approx. 53.49% at −1.78% compressive strain, showing superior strain detection ability.

The gauge factor is the key parameter describing the sensitivity of strain sensors and can be calculated using the following formula [25]:


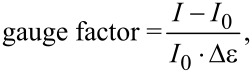


where *I* is the current in the compressive state or in the strained state and *I*_0_ is the current when the sample is not strained. It can be seen from Figure 3d that, regardless of the strain, the gauge factor shows an increasing trend at a bias voltage of 1.9 V. At a strain of −1.78% (compressive strain) or 1.78% (tensile strain), the gauge factor is as high as 30, which enables the detection of a very small deformation. Compared with previous works [27–29], the AlGaN/AlN/GaN NW-based strain sensor has a higher sensitivity. The compressive strain and the tensile strain are converted to a normal strain ε by using the following formula [25–26]:


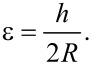


Here, the PET substrate was bent to a radius *R* and the thickness *h* is based on the assumption that the strain of the PET substrate is considered as the strain of the AlGaN/AlN/GaN NW-based strain sensor.

**Figure 4:**
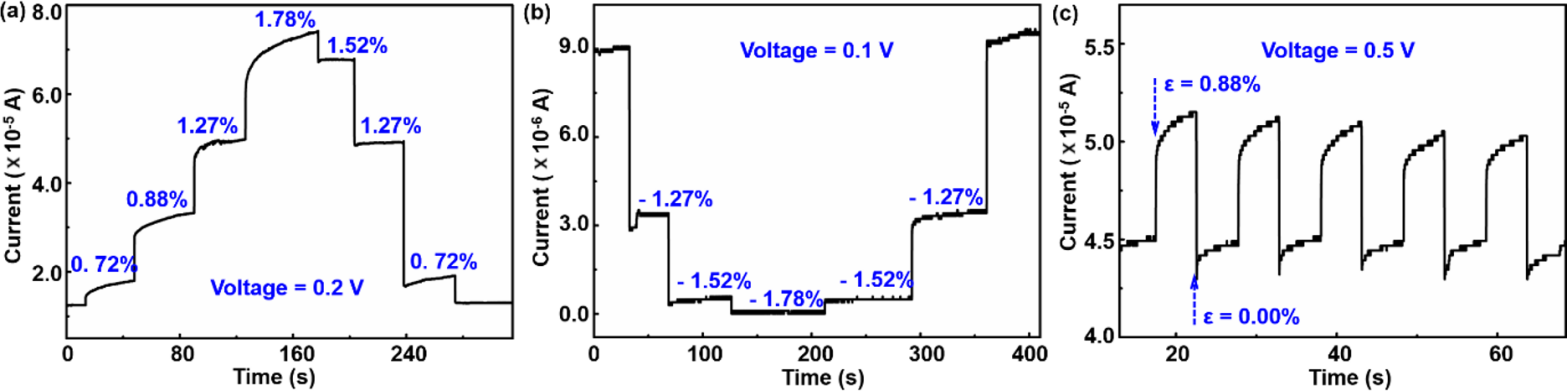
(a) *I*–*t* characteristic curve at a bias voltage of 0.2 V under tensile strain. (b) *I*–*t* characteristic curve at a bias voltage of 0.1 V under compressive strain. (c) *I*–*t* characteristic curve under periodic tensile straining to 0.88%.

The stability and repeatability of the AlGaN/AlN/GaN NW-based strain sensor is demonstrated in Figure 4. Tensile and compressive stress was repeatedly applied and released. At a bias voltage of 0.2 V, the output current increased with the increase in the tensile strain. The output current went back to the initial value when the stress was continuously released (Figure 4a). Conversely, at a bias voltage of 0.1 V, the output current continuously decreased until a −1.78% compressive strain value was reached. Then, it gradually returned to the initial value (Figure 4b). The results show that the strain influences the transmission of carriers and the output current signal even under small bias voltage and strain values, which further demonstrates the sensitivity and stability of the strain sensor. A test with five cycles of repetitive mechanical load and unload was carried out, as shown in Figure 4c. The results demonstrate that the strain sensor still has a superior performance after multiple cycles at 0.88% tensile strain, which indicates its stability.

**Figure 5:**
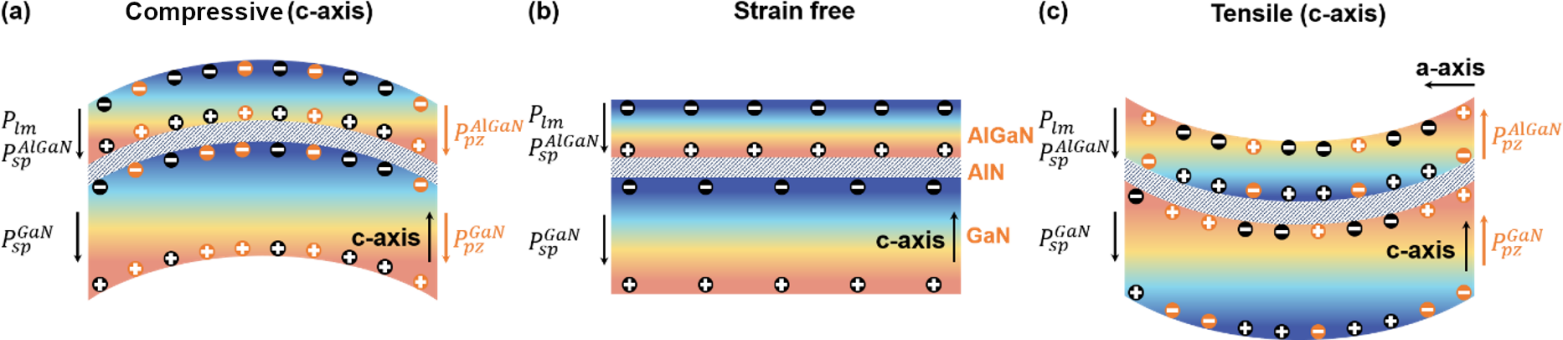
Structure diagram showing the charge distribution of the AlGaN/AlN/GaN heterojunction NW under compressive strain (a), without strain (b), and under tensile strain (c). The black color represents the inherent polarization and the charge generated by the inherent polarization, whereas the orange color represents the piezoelectric polarization and the charge generated by the piezoelectric polarization.

The polarization charge distribution of the AlGaN/AlN/GaN heterojunction NW under different strain conditions is shown in Figure 5. This model depicts the working mechanism of the strain sensor. Due to the non-centrosymmetric structure of nitride semiconductor materials, *P*_sp_ and *P*_lm_ can occur. The direction of polarization (shown in Figure 5b), 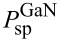, 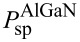, and *P*_lm_ are along the negative direction of the *c*-axis. That is, the positive polarization charges are gathered on the −*c* plane whereas the negative polarization charges are generated on the +*c* plane. At the heterojunction interface, the positive polarization charge on the bottom surface of AlGaN couples with the negative polarization charge on the top surface of GaN, resulting in a positive net polarization charge. This results in the attraction of free electrons in the GaN layer, which gather on the GaN side and form a 2DEG [4]. The 2DEG at the heterojunction interface has a higher electron mobility (1.2 × 10^3^ cm^2^·V^−1^·s^−1^) and sheet density (8.6 × 10^12^ cm^−2^), values obtained from the Hall test, which greatly improves the performance of the AlGaN/AlN/GaN heterojunction NW-based strain sensor.

In Figure 5a, the AlGaN/AlN/GaN heterojunction NW under compressive strain (c-axis) generated a piezoelectric polarization along the negative direction of the *c* axis, which resulted in a negative piezoelectric charge on the +*c*-plane of the GaN layer. The corresponding net polarization charge at the heterojunction interface decreases, resulting in a decrease in the density of the 2DEG. Therefore, as the compressive strain (c-axis) increases, the output current gradually decreases, which is consistent with the experimental results obtained (Figure 3). On the contrary, under tensile strain (*c*-axis), the direction of the piezoelectric polarization is along the positive direction of the *c*-axis, and positive piezoelectric polarization charges are generated on the +*c*-plane of GaN. Therefore, the net polarization charge at the heterojunction interface increases, resulting in an increase in the density of the 2DEG and a corresponding increase in the output current (Figure 5c and Figure 3). The results show that mechanical strain changes the concentration of the 2DEG at the heterojunction interface by changing the polarization charge distribution. This, in turn, influences the output current signal of the piezotronic strain sensor.

## Conclusion

AlGaN/AlN/GaN heterojunction NWs with controllable size were prepared by a top-down two-step process, including ICP dry etching and selective EC wet etching. After the lift-off, a single NW was transferred to a flexible PET substrate and was fixed by ITO electrodes to form an ohmic contact for the strain sensor. We have introduced the piezotronic effect to adjust the carrier transmission and the output current under the action of an applied stress. The output current increased with an increasing tensile strain, decreased with an increasing compressive strain, and went back to the initial value after the release of either the compressive or the tensile strain, which shows the strain detection ability of the device. The gauge factor was calculated under different strain conditions. It was as high as 30 under either a −1.78% compressive strain or a 1.78% tensile strain, which shows the high sensitivity of the sensor. Furthermore, multiple cycles of tensile stress–release testing also fully demonstrated the repeatability and stability of the strain sensor. The working principle model of the strain sensor was illustrated by the polarized charge distribution under compressive or under tensile strain. This work describes a highly sensitive and a highly stable strain sensor based on a new AlGaN/AlN/GaN NW structure, which has shown great application potential in several fields, including wearable integrated circuits, health-monitoring devices, and artificial intelligence.

## Experimental

### Synthesis of the epitaxial structure

The epitaxial structure used in this study was grown by MOCVD (Thomas Swan). Trimethylgallium (TMGa), trimethylaluminum (TMAl), and ammonia (NH_3_) were used as Ga, Al, and N sources, respectively. N_2_ and H_2_ were used as carrier gases in the growth process. A 1 μm layer of unintentionally doped GaN was deposited as the buffer layer on a sapphire substrate, followed by a 500 nm layer of Si-doped N-GaN (the doping concentration was 5 × 10^18^·cm^−3^). The thickness of the heavily doped GaN was 1.5 μm and the Si doping concentration was 1.0 × 10^19^·cm^−3^. The thickness of the two thin N^++^-GaN layers was only 10 nm each with a Si concentration of 4.5 × 10^19^ cm^-3^. The thickness values of U-GaN, U-AlN, and U-Al_0.3_Ga_0.7_N were 900 nm, 1.5 nm, and 30 nm, respectively.

### Preparation of the AlGaN/AlN/GaN NW-based strain sensor

First, stepper lithography was used to form NW stripes with a controllable size. Here, we set the stripe width to 900 nm and the stripe spacing to 3 μm. Then, the wafer was etched by ICP. The etching depth reached the heavily doped GaN to expose the sacrificial layer for EC wet etching. Next, the wafer was laser-cut into a rectangular sample, where the NWs were aligned parallel to the long sides. One end of the sample was coated with a silver paste and connected to the anode of the electrolytic cell, and the cathode was the Pt sheet. Oxalic solution (0.3 M) was used as the electrolyte. The applied voltage was 20 V and the duration of its application was 10 min. After selective EC wet etching, the sample with suspended NWs was placed in deionized water to remove the etching residues. Then, the cleaned and dried NWs were electrostatically adsorbed onto a PET substrate in order to print and fix on the pre-cured host substrate, such as another PET substrate or room-temperature-vulcanizing silicone (RTV) substrate. Herein, a single AlGaN/AlN/GaN NW was transferred to a flexible PET substrate, and ITO electrodes were prepared by magnetron sputtering on both ends of the NW to form an ohmic contact.

### Measurements

The selective EC etching process and the morphology of the NWs were imaged using an optical microscope (Leica DM2500M), an SEM (ZEISS Ultra 55), and a TEM (JEM-2100HR, JEM-1400 PLUS). The *I*–*V* characteristic curves were measured using a source table including a SR570 low-noise current preamplifier and a DS345 function generator.
